# Catheter-based polarimetric imaging to complement MRI for deep brain stimulation neurosurgery

**DOI:** 10.1117/1.NPh.12.3.035001

**Published:** 2025-07-25

**Authors:** Shadi Masoumi, Maxina Sheft, Mireille Quémener, Alexandre Bédard, Valérie Pineau Noël, Martin Parent, Martin Villiger, Daniel C. Côté

**Affiliations:** a Université Laval, CERVO Brain Research Center, Québec, Canada; b Université Laval, Centre d’Optique, Photonique et Laser (COPL), Québec, Canada; c Harvard Medical School, Boston, Massachusetts, United States; d Massachusetts General Hospital, Wellman Center for Photomedicine, Boston, Massachusetts, United States; e Massachusetts Institute of Technology, Institute for Medical Engineering and Science, Cambridge, Massachusetts, United States

**Keywords:** polarization-sensitive optical coherence tomography, nonhuman primates, Parkinson’s disease, white matter fiber tract, birefringence

## Abstract

**Significance:**

Deep brain stimulation (DBS) is an established treatment for movement disorders and other neurological conditions. Accurate localization of small deep brain nuclei, such as the subthalamic nucleus (STN) and internal pallidum (GPi), is crucial for successful DBS outcomes. However, magnetic resonance imaging (MRI), commonly used for DBS planning, lacks the resolution and contrast needed to directly delineate these target structures.

**Aim:**

We aim to explore the potential of catheter-based polarization-sensitive optical coherence tomography (PS-OCT) as a complementary imaging tool for high-resolution visualization of tissue surrounding the DBS insertion trajectory.

**Approach:**

We simulated DBS implantation surgery at three targets in a post-mortem nonhuman primate head. PS-OCT, using advanced reconstruction algorithms for absolute depth-resolved birefringence, was compared with MRI for its ability to visualize and differentiate structural details.

**Results:**

PS-OCT provided more detailed and accurate structural information than MRI while maintaining consistency with MRI results. Its compact form factor and imaging paradigm integrate seamlessly into the surgical workflow, offering new insights for intraoperative decision-making.

**Conclusions:**

PS-OCT functions as an intraoperative imaging tool, offering valuable guidance during the procedure. These findings establish PS-OCT as a promising complementary tool for DBS, with potential for further clinical validation and *in vivo* studies.

## 1 Introduction

Annually, around 23 million people worldwide suffer from brain disorders, with 14 million requiring surgery for issues such as brain tumors, traumatic brain injuries, and neurodegenerative diseases such as Parkinson’s disease (PD).^1^ Our focus is on deep brain stimulation (DBS), a stereotactic neurosurgical procedure that involves implanting electrodes in specific brain regions to regulate abnormal neural activity.^2^ This technique, introduced by Benabid,^3^ is primarily used to treat movement disorders such as PD, dystonia, and essential tremor. MRI is an essential part of neurosurgical planning, where a pre-operative MRI (pre-op MRI) of the patient’s head is used to identify critical targets. DBS targets vary according to the disorder being treated: for instance, the subthalamic nucleus (STN) and internal pallidum (GPi) are commonly targeted for PD, the ventral intermediate thalamic nucleus (VIM) for essential tremor, and the caudate nucleus (CD) and the internal capsule (IC) for severe forms of obsessive-compulsive disorder (OCD).^4^^–^^7^ Once the target of interest is identified in MR images, its stereotactic coordinates are determined relative to anatomical landmarks such as the anterior and posterior commissures and to the stereotaxic frame placed on the patient’s head. After identifying the target, final adjustments and confirmation of the target may be performed in the operating room through microelectrode recording (MER) and macrostimulation when available.^5^

Clinical outcomes in DBS depend heavily on the precise placement of stimulation electrodes. Lack of efficiency in nearly 50% of unsuccessful DBS surgeries is due to incorrect lead placement and improper targeting and can be rescued by repositioning the lead by as little as 1 mm.^8^^–^^11^ There remain significant challenges in DBS target localization, surgical planning, and electrode implantation. One major challenge is that conventional MRI techniques often lack the contrast and resolution needed to clearly visualize small, deeply located structures and rely on mapping the MRI to anatomic brain atlases, making accurate targeting difficult. Recently, diffusion magnetic resonance imaging (dMRI)-assisted DBS has shown promise by visualizing brain white matter (WM) fiber pathways.^12^^,^^13^ However, it is expensive and its use is often confined to well-funded, specialized centers. In addition, intraoperative brain shift caused by factors, such as cerebrospinal fluid loss upon cranial opening, inherent variability in brain anatomy, stereotactic frame misalignment, and limited real-time feedback during surgery, particularly when intraoperative MRI is not used, further reduces neurosurgical accuracy. Although intraoperative MRI can be useful, it presents its own challenges, including high cost, limited availability, the need for specialized equipment and trained personnel, and a time-consuming procedure. As a result, the only intraoperative tool for providing feedback during neurosurgery is MER. However, it offers only discrete electrophysiological data along the insertion path, lacking continuous structural information, and is often performed only along the distal portion of the insertion path rather than the entire trajectory. Furthermore, MER is only available when patients are awake and for specific indications that allow real-time feedback. To address these challenges, optical imaging techniques with significantly better contrast than MRI, higher resolution, and lower costs are being explored as valuable supplementary tools in intraoperative DBS clinical practice. These include diffuse reflectance spectroscopy,^14^^–^^17^ laser Doppler flowmetry,^18^^,^^19^ Raman spectroscopy,^20^^,^^21^ optical coherence tomography (OCT),^22^^–^^24^ and polarization-sensitive optical coherence tomography (PS-OCT).^25^ Among them, OCT offers significant advantages due to its ability to produce high-resolution cross-sectional images owing to its interferometric depth encoding without the need for depth scanning. Furthermore, the integration of probes or catheters allows imaging in hard-to-reach areas. The size of existing OCT catheters closely matches that of DBS leads, making catheter-based OCT an ideal fit for assisting stereotactic neurosurgical procedures. However, the high scattering of brain tissue makes it challenging to differentiate tissue types or extract detailed microstructural information. By utilizing the polarization properties of light, PS-OCT can provide additional contrast beyond conventional OCT signals of scattering intensity. Specifically, PS-OCT captures the intrinsic characteristic of brain WM known as birefringence. In birefringent materials such as WM, light encounters two slightly different refractive indices depending on whether the light is polarized parallel or perpendicular to the tissue fiber direction, a property known as optic axis (OA) orientation. Polarized light imaging and PS-OCT have been extensively studied for investigating WM fiber bundle alignment and structure.^25^^–^^28^ In addition, some studies have explored the application of PS-OCT for brain imaging in comparison with dMRI,^29^^–^^31^ and it has been utilized to enhance the precision of dMRI reconstruction algorithms.^32^^,^^33^ In our own previous research toward DBS guidance,^25^ small-diameter catheters and advanced processing algorithms were employed to obtain depth-resolved orientation of WM fiber bundles in a fresh porcine brain. The performance of the catheter-based PS-OCT system was validated by comparison with a well-characterized benchtop PS-OCT system, which served as a reference standard for accurate birefringence measurements. The results suggested that catheter-based PS-OCT could help in optimal electrode placement during DBS using white matter fiber orientation as an additional source of guidance.^25^ Given its abilities, catheter-based PS-OCT demonstrates significant potential to improve guidance in neurosurgical procedures and integrate with standard MRI-based planning. Birefringence, the optical property measured by PS-OCT, reflects the tissue’s structural anisotropy. The amount of birefringence detected in tissue is directly related to the alignment and density of the fibrous structures within it. Highly organized and densely packed fibers of WM regions produce significant birefringence, which is captured by PS-OCT, akin to the diffusion anisotropy measured by diffusion-weighted MRI. This information is essential for accurate targeting in DBS neurosurgery as it enables the identification of critical WM tracts that can serve as landmarks. Here, we present, for the first time, the results of catheter-based PS-OCT and MRI imaging on a fresh post-mortem monkey head, focusing on three primary DBS targets. We demonstrate how advanced polarization-sensitive reconstruction algorithms provide more detailed and accurate information than MRI while remaining consistent with MRI findings. This allows for enhanced visualization of brain structures that are difficult to resolve with conventional imaging, making it particularly useful for DBS neurosurgery, where precise targeting is crucial.

## 2 Materials and Methods

### 2.1 Catheter-Based PS-OCT System and Processing

All PS-OCT endoscopic imaging was carried out using the NvisionVLE Imaging System (Soléron LLC, Marlow, New Hampshire, United States), which was adapted for polarization-sensitive imaging. The system’s wavelength-swept light source operated at a central wavelength of 1300 nm, with an A-line repetition rate of 50 kHz and a wavelength scanning range of 110 nm. This configuration resulted in a depth resolution of ∼9  μm in tissue. A fiber-based electro-optic modulator was used to generate alternating polarization states, orthogonal on the Poincaré sphere, for subsequent A-lines. The horizontal and vertical polarization components of the backscattered light were captured with polarization-diverse detection. The rotating catheter is made of a fiber probe enclosed within a protective transparent polymer sheath with an outer diameter of 2.3 mm, enabling imaging through a helical scan pattern created by simultaneous rotation and pullback of the probe. The probe features a graded-index lens and a deflecting prism at the distal tip to direct light toward the sample and optimize collection, achieving a lateral resolution of 40  μm. The system allowed flexibility in terms of pullback speed, as well as the number of A-lines and B-scans. Data were acquired at ∼25 frames per second, corresponding to 25 full probe rotations per second, with each frame consisting of 2048 A-lines. The pullback speed was set to 5  mm/s, resulting in a catheter rotation rate of 1500 revolutions per minute (RPM). This configuration yielded a helical pitch of 0.2 mm per rotation. The angular sampling density was ∼0.176  deg per A-line. A single pullback was performed per trajectory, without averaging across multiple acquisitions. In addition to the intensity tomograms that can be acquired using conventional OCT systems, the polarization-sensitive system allows the extraction of depth-resolved birefringence properties of the sample, including local retardance and depth-resolved OA orientation, as described thoroughly in Ref. 25. In short, using the Stokes formalism and describing retardance as a rotation of the Stokes vectors on the Poincaré sphere, we first retrieved the cumulative retardance/rotation matrix of the sample, which accounts for the combined effect of the sample, system components, and the rotating catheter on the input polarization states. Taking advantage of the round-trip PS-OCT measurements and the tissue being linearly retarding, we compensated for the catheter and system transmission effects.^34^ In addition, wavelength-dependent polarization effects from static system components and the effects of speckle were mitigated through spectral binning and Gaussian filtering, respectively.^35^ Notably, dynamic rotation of the catheter introduces varying OA offsets. In our previous catheter-based PS-OCT study of the brain, this issue was addressed by modeling the effect of the catheter transmission.^25^ In our most recent work on optic axis reconstruction for catheter-based PS-OCT, we presented the guide star method, where the birefringence properties of the protective catheter sheath are used directly to correct for these varying OA offsets and obtain absolute OA with a three-fold improvement compared with our earlier modelling method.^36^ Finally, after obtaining the correct OA using the guide star approach, depth-resolved birefringence and absolute OA orientation reconstruction were achieved by correcting for the effects of preceding linearly retarding tissue layers, each one pixel thick.

### 2.2 MRI System

All 3 Tesla T1w scans were acquired using the Siemens MAGNETOM Skyra system. The sequence parameters were as follows: echo time (TE) of 3.89  μs, repetition time (TR) of 2300  μs, flip angle of 9 deg, and a field of view of 256 mm. The acquisition matrix was set to 256×256, with an averaging factor of 9, resulting in a voxel resolution of $0.57\times 0.57\times 0.57\;{\mathrm{mm}}^{3}$.

### 2.3 Tissue Sample

The experiment was conducted on the head of an adult female cynomolgus monkey (*Macaca fascicularis*; age, 3.9 years; weight, 3.8 kg). The animal was euthanized at 9:15 AM, after which the head was promptly removed and preserved in phosphate-buffered saline (PBS) until the procedures began later that day at 3 PM. Experiments were approved by the Comité de Protection des Animaux de l’Université Laval, in accordance with the Canadian Council on Animal Care’s guide to the Care and Use of Experimental Animals.

### 2.4 Surgical Procedure

The experiment aimed to closely mimic a standard DBS procedure, with the key difference being the use of a PS-OCT probe instead of a conventional DBS electrode. We targeted three basal ganglia nuclei to validate our approach: STN, external pallidum (GPe), and CD, all in the left hemisphere. The stereotactic coordinates for the centers of these nuclei were obtained from a standard macaque brain atlas^37^: STN (AP, 12.1 mm; ML, 5 mm), GPe (AP, 17.2 mm; ML, 6 mm), and CD (AP, 14.6 mm; ML, 11 mm). For ease of analysis, we selected parallel, vertically aligned trajectories for probe insertion in the dorsoventral direction. Following a pre-op MRI scan, the head was secured in a Model 1430 stereotaxic frame (Kopf Instruments, Tujunga, CA, United States), and a $1\times 1\;{\mathrm{cm}}^{2}$ section of the cranium was opened. The dura mater was then removed to allow direct access to the brain, and the PS-OCT probe was guided with a micromanipulator stereotaxic holder along the vertical axis. Measurements were collected in the left hemisphere according to the planned coordinates for the three nuclei. The probe was inserted into the target depth, and data acquisition was performed over a pullback length of ∼23  mm from the basal ganglia targets toward the cerebral cortex. Each trajectory took ∼6  s to complete, recording 115 B-scans over the 23 mm pullback. To preserve tissue integrity during measurements, the brain surface was regularly moistened with PBS. After completing the PS-OCT measurements, capillary tubes filled with vitamin E were inserted along the same stereotactic paths. These rods extended beyond the PS-OCT pullback range, leaving visible insertion tracks in the tissue and serving as landmarks for subsequent MRI imaging. Following these procedures, the head was stored in PBS at 4°C. A post-operative MRI (post-op MRI) scan was performed the next day, using the same T1w sequence as for the pre-op MRI scan.

### 2.5 Post-operative Tissue Handling and Brain Section Analysis

On post-mortem day 2, the brain was extracted from the skull. The left hemisphere was fixed by immersion in 4% paraformaldehyde at 4°C for 72 days and then sliced into 400  μm transverse sections using a freezing microtome. A top-view photograph of the tissue block was taken each time a section was collected. Images with visible insertion tracks were retained for further analysis and served as the initial ground truth for tissue classification, which was later compared with PS-OCT data.

### 2.6 Co-registration between MRI and PS-OCT

**Fig. 1:**
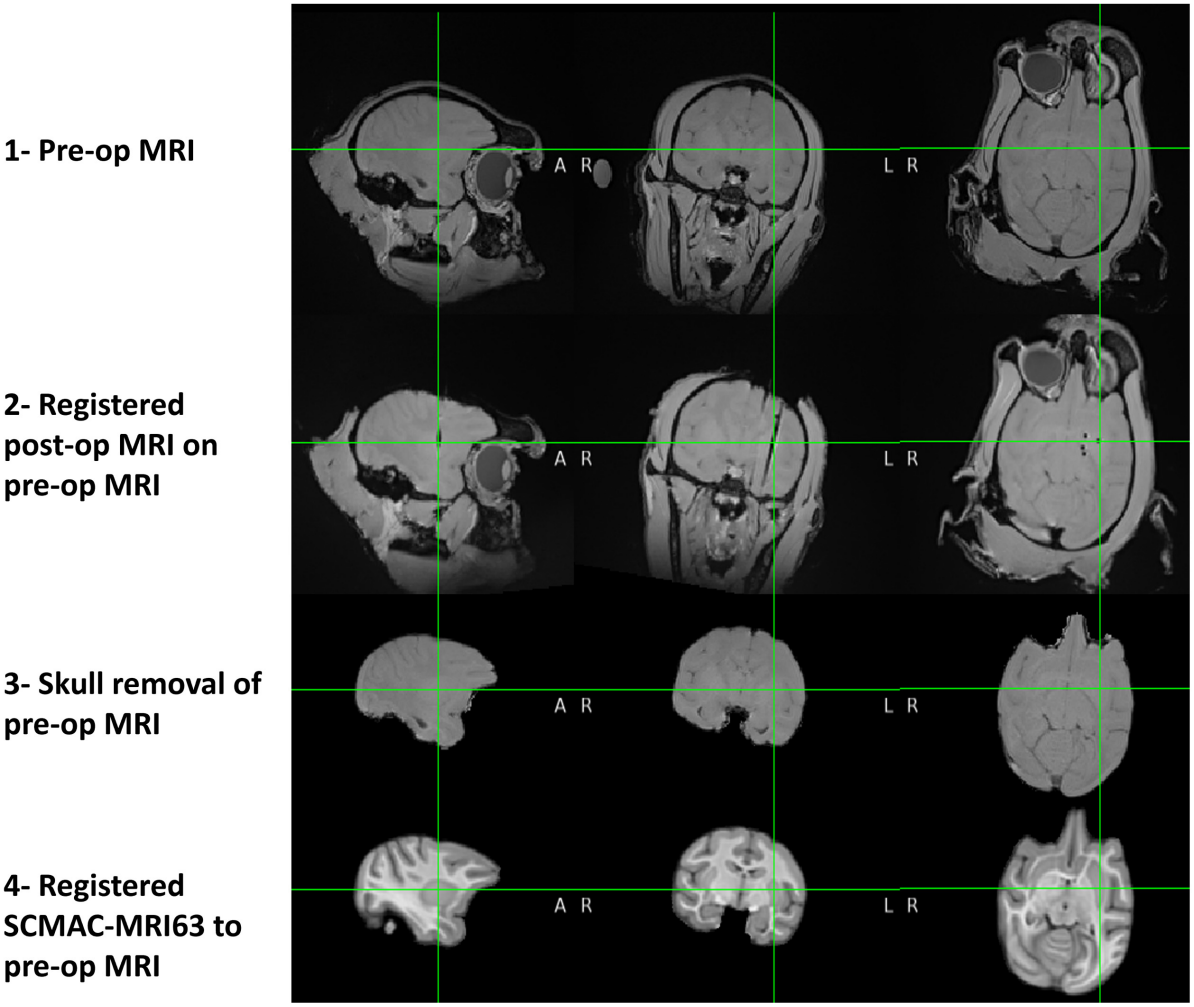
Overview of the skull removal and registration process, aligning the post-op MRI to the pre-op MRI to determine the imaging trajectory coordinates in the pre-op MRI scan, followed by the registration of the SCMAC-MRI63 template to the pre-op MRI from the CERVO-MRI dataset.

The final step in co-registration involved mapping the PS-OCT imaged volume to the corresponding region in the pre-op MRI data, though several prerequisite steps must be completed beforehand, as depicted in Fig. 1. First, we registered the post-op MRI data to the pre-op MRI to determine the imaging trajectory coordinates in the pre-op MRI scan, on which brain tissue was intact. All MRI registrations in this study were performed using FSL’s affine registration, 12-parameter model,^38^^,^^39^ followed by ANTs,^40^ applying a sequence of rigid, affine, and SyN transformations across four scales with histogram matching and intensity winsorization to reduce contrast variability. Due to the limited contrast of the 3T MRI scans obtained from the post-mortem monkey head, we registered a high-quality T1w MRI template constructed from 63 male cynomolgus monkeys (SCMAC-MRI63)^41^ to our pre-op MRI dataset (see specifications in Table 1, where CERVO-MRI refers to the original 3T MRI scans performed in this study). Contrast enhancement techniques were considered but found insufficient due to the inherently low signal quality of the post-mortem MRI, prompting the use of the template to improve anatomical reference and visualization.

**Table 1:** MRI scan parameters for our CERVO-MRI datasets and SCMAC-MRI63 template.

Parameter	CERVO-MRI	SCMAC-MRI63
Species	*Macaca fascicularis*	*Macaca fascicularis*
Numbers	1	63
Sex	Female	Male
Age	3.9 years	4.86 years, std: 0.56
Weight	3.8 kg	Mean: 5.05 kg, std: 0.33
System	3.0 T scanner, Siemens Healthineers (Erlangen, Germany), Magnetom Prisma	3.0 T scanner (Siemens Trio, Siemens Medical Solutions, Malvern, Pennsylvania, United States)
Repetition and echo times	TR: 2300 μs, TE: 3.89 μs	TR: 2000 μs, TE: 2.98 μs
Flip angle	9 deg	12 deg
Field of view	256 mm	128 mm
Matrix	256 × 256	128 × 128
Number of averages	9	2
Voxel resolution	$0.57\times 0.57\times 0.57\;{\mathrm{mm}}^{3}$	$1.0\times 1.0\times 1.0\;{\mathrm{mm}}^{3}$

To optimize registration accuracy, skull stripping was performed using a combination of FSL and ANTs. The registration pipeline followed the same steps and criteria as those used for aligning the post-op MRI to the pre-op MRI, utilizing both FSL and ANTs tools. Registration quality was evaluated using normalized cross-correlation (NCC) and normalized mutual information (NMI) metrics, which confirmed accurate spatial alignment. Specifically, the NCC and NMI values between the post-op and pre-op MRIs were 0.9918 and 0.7424, respectively, whereas those between the SCMAC-MRI63 and the pre-op MRI were 0.9717 and 0.6523, respectively. In addition, visual inspection of anatomical landmarks was performed to ensure anatomical consistency. Although the NMI value between the SCMAC-MRI63 and pre-op MRI appears relatively low, this is expected due to the limited contrast in the pre-op scan. Ultimately, this process provided a high-quality T1w MRI of the same monkey species, within which the coordinates of the PS-OCT imaged volume were precisely located.

**Fig. 2:**
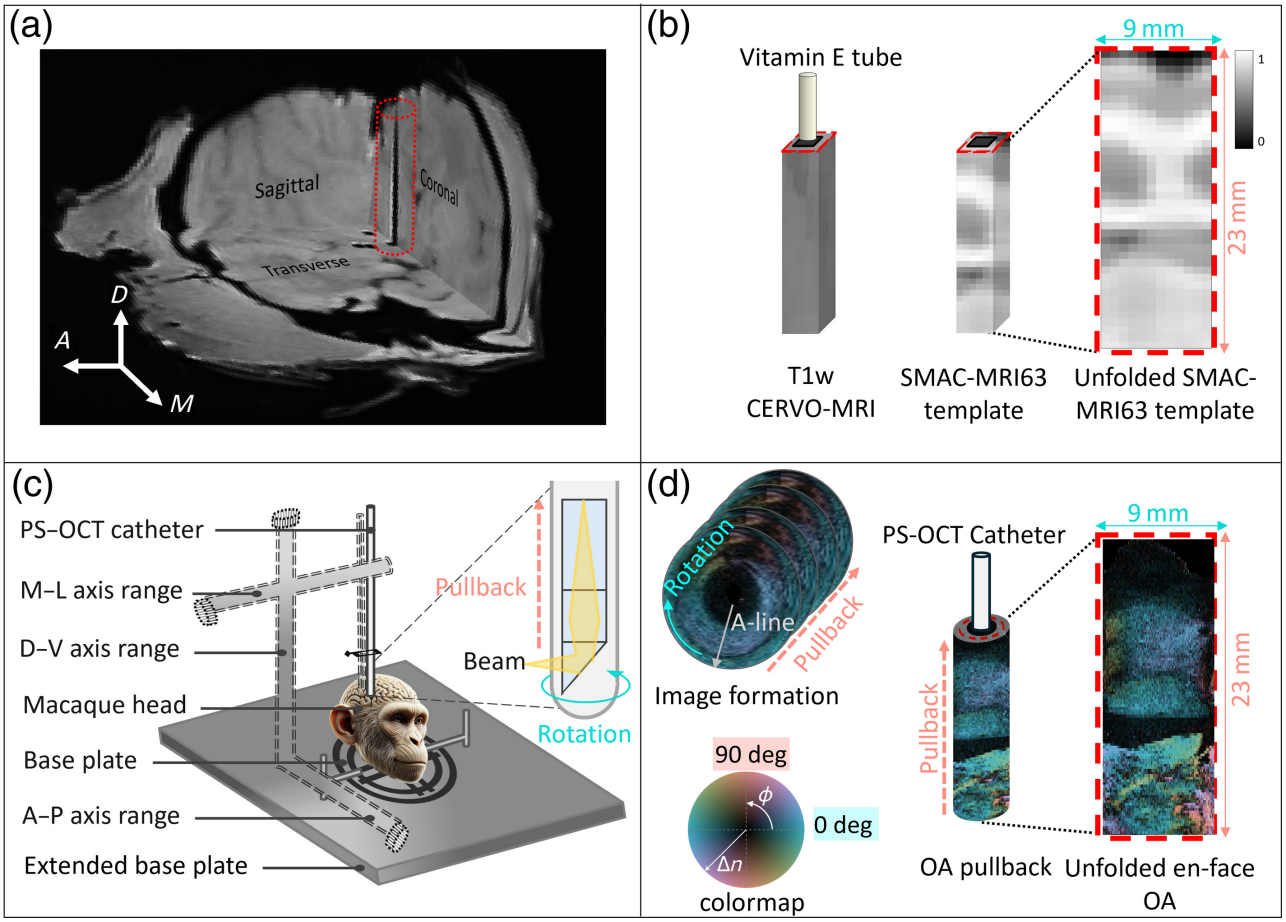
Co-registration of PS-OCT and MRI data. (a) The imaged track is displayed in a CERVO-MRI T1w scan, visualized in three dimensions. (b) The extraction of the imaged volume from the T1w CERVO-MRI scan is shown, along with the corresponding volume from the SCMAC-MRI63 template. (c) A stereotactic frame with an attached PS-OCT catheter is depicted, illustrating its positioning during imaging, along with a magnified view of the catheter tip. (d) The PS-OCT image formation process is illustrated, showing the acquisition of a single A-line, the formation of rotations and one pullback, as well as the unfolded *en face* PS-OCT image (carpet view) with OA colormap.

Next, to extract the MRI signal corresponding to the tissue volume imaged by PS-OCT, we defined a cube-shaped region of interest (ROI) within the MRI volume along the three catheter trajectories. Each cube cross-section included nine central pixels representing the approximate catheter diameter and insertion path, surrounded by 16 neighboring pixels. This pixel configuration was selected to match the native MRI resolution (0.57×0.57×0.57  mm). The 16 surrounding pixels covered ∼9  mm, corresponding to the circumference of the PS-OCT carpet view, which originates from an imaging depth about 300  μm outside the catheter sheath. The length extended from the cortical surface to the deep brain targets, covering at least 21 mm, which corresponds to 105 PS-OCT slices and 37 MRI slices. We then unfolded the cube shape into a two-dimensional representation for better visualization [Fig. 2(b)]. We also converted each PS-OCT pullback into a two-dimensional unrolled *en face* image (carpet-view), representing the same tissue as the co-registered MRI-unfolded cube shape. The MRI data were rigidly registered to the PS-OCT coordinate space, and the ROI was mapped accordingly without any resampling or averaging, thereby preserving the original spatial resolution of both datasets. Figure 2(d) illustrates the depth-resolved OA visualization along with its colormap. In this colormap, OA orientation is represented by color hue, overlaid by birefringence as brightness. As regions without birefringence exhibit random OA orientations, both OA orientation and scalar birefringence are displayed together. The color blue (0 deg) on the colormap indicates that the fiber tracts are oriented circumferentially, perpendicular to the catheter or imaging trajectory, considering that brain WM is negatively birefringent.^42^ This interpretation is consistent with our previous work, in which a birefringent phantom with a known and constant OA orientation, exhibiting positive birefringence, appeared blue when its OA orientation aligned with the catheter.^36^ The change in the sign of the birefringence results in an apparent flip of the corresponding OA orientation.

## 3 Results

**Fig. 3:**
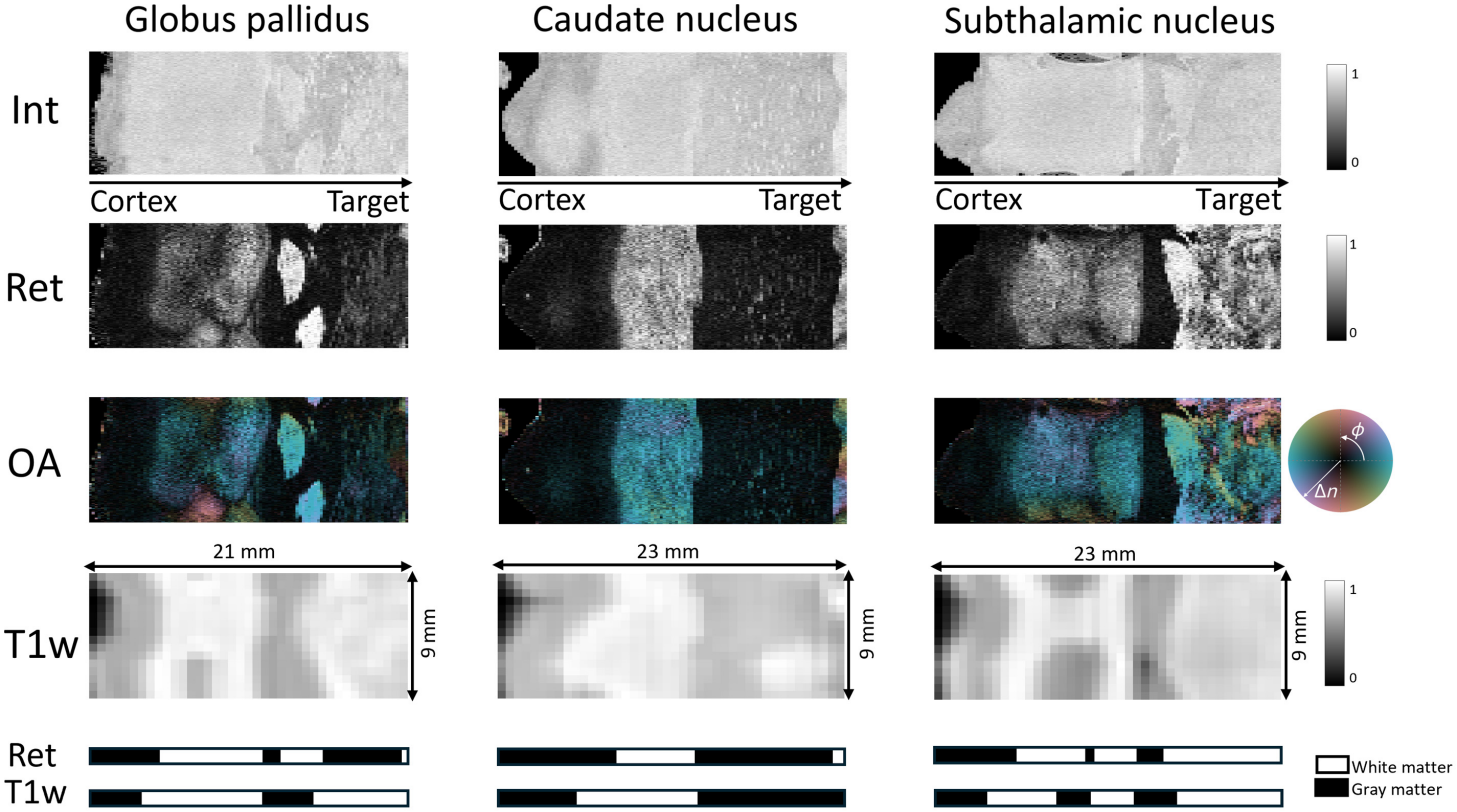
PS-OCT and T1w MRI carpet views for three target nuclei. From top to bottom, each column shows Int, Ret, OA orientation, T1w MRI from the SMAC-MRI63 template registered to pre-op MRI, and the corresponding binary tissue barcodes derived from the PS-OCT retardance and T1w profile. Colorbars for each modality are shown on the right.

Here, we present the results of deep brain imaging along three distinct tracks targeting the STN, CD, and GPe. All three imaging trajectories covered at least 21 mm (equivalent to 105 PS-OCT B-scans) with consistent data quality from the target to the cerebral cortex. No dropouts or obvious nonuniform rotational distortions were observed. The head and the probe were securely attached to the stereotactic frame, and we took great care in handling the probe to avoid bending or misalignment. In addition, the ability to visualize the trajectory through real-time intensity images was helpful in ensuring accurate and high-quality data acquisition. Figure 3 shows PS-OCT intensity (Int), scalar local retardance (Ret), and OA orientation images alongside the SMAC-MRI63 template T1w of the imaged volume for the STN, CD, and GPe tracks. In general, both T1w MRI and PS-OCT agree in distinguishing tissue types. However, PS-OCT excels at identifying finer structures due to its micrometer-level resolution, which is much higher than the millimeter resolution of MRI. As anticipated, WM exhibits stronger scattering compared with GM regions such as the cerebral cortex. The difference between WM and GM is even more pronounced in retardance images, where WM shows significantly higher birefringence due to its fibrillar structure. In addition, the OA orientation of PS-OCT offers insight into the direction of WM tracts, by indicating the azimuthal orientation relative to the OCT imaging beam. In the subcortical WM of the CD trajectory, we observed significantly higher birefringence compared with regions such as the STN or GPe. The lower birefringence in these latter areas can likely be attributed to several factors. One possibility is the presence of densely interwoven fiber tracts, which are below the resolution of the system, making it difficult for PS-OCT to detect distinct birefringence patterns. Another contributing factor could be the relative alignment of the fibers with the OCT imaging beam. The highest birefringence is observed where the fibers are orthogonal to the beam. Fibers parallel to the beam do not result in any measurable birefringence. To facilitate comparison between PS-OCT and MRI data, we implemented a simplified segmentation approach based on the 1D profiles. Specifically, the profile was extracted along the imaging trajectory by averaging across image rows, followed by k-means clustering (k=2) to separate the data into two tissue types, WM and GM. The midpoint among the cluster centers was used as a threshold to generate a binary segmentation, which we refer to as a tissue barcode. Figure 3 includes this barcode for both the PS-OCT retardance data and the T1w MRI, providing a compact visual representation of tissue transitions along the probe path.

It is worth noting that a slight co-registration error between MRI and PS-OCT is anticipated, although its magnitude is expected to be minimal. Importantly, we did not perform direct image–based registration between PS-OCT and MRI. Instead, we co-registered the OCT catheter trajectories based on the position of capillary tubes filled with vitamin E for MRI visualization, which were placed along the insertion trajectories left by the retracted PS-OCT catheter. Two main sources contribute to a possible registration error: (1) inaccuracies in MRI registration used to estimate the imaging trajectory within the pre-op MRI and its transformation to the SMAC-MRI63 template, particularly due to the limited contrast of the pre-op scan, and (2) general experimental inaccuracies, such as placement of the vitamin E markers and the PS-OCT acquisition process. To address the first source, we validated the MRI registration pipeline using quantitative metrics, including NCC and NMI, which confirmed robust alignment. Specifically, the NCC and NMI values between the post-op and pre-op MRIs were 0.9918 and 0.7424, respectively, whereas those between the SMAC-MRI63 and the pre-op MRI were 0.9717 and 0.6523, respectively. In addition, the SMAC-MRI63 template is an average of 69 male cynomolgus monkeys of varying ages and weights, which may contribute to minor anatomical differences. As for the second source of error, careful handling and placement of both the probe and the capillary tubes, along with secure fixation within the stereotactic frame, minimized the likelihood of major deviations. However, these experimental errors are difficult to quantify precisely. Furthermore, regardless of co-registration error, the limited resolution of MRI can result in the partial volume effect, where signals from different tissue types such as WM and GM are mixed within a single voxel.^43^ This reduces the contrast between tissue types and makes it harder to distinguish fine anatomical details. As a result, PS-OCT is able to detect structural details that are beyond MRI’s capabilities.

**Fig. 4:**
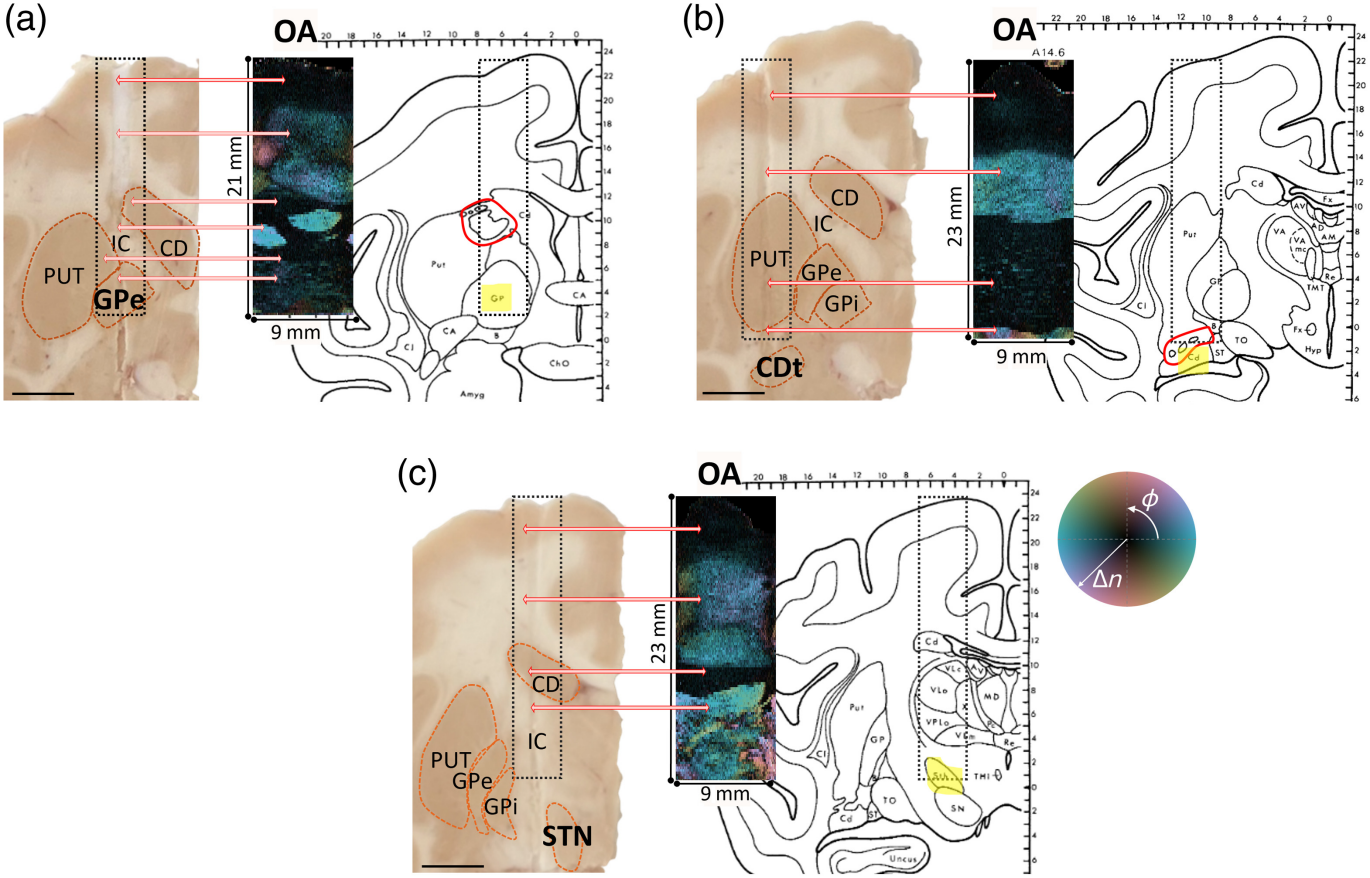
Tissue photographs, corresponding brain atlas sections, and the OA carpet views for three distinct trajectories: (a) GPe, (b) CD, and (c) STN. Dashed black rectangles highlight the imaged regions, whereas red outlines mark specific WM anatomical landmarks in the brain atlas. The yellow shaded areas denote the target regions. The scale bar represents 5 mm, and the annotated tissue structures are as follows: CD, caudate tail (CDt), GPe, GPi, IC, PUT, and STN. The two-way red arrows indicating spatial correspondence between the tissue photographs and OA maps.

Figure 4 compares the PS-OCT trajectories with the anatomical information from the tissue slice and the macaque brain atlas^37^ that was used for stereotactic planning. Although the OA data and MRI carpet views are more representative of the underlying volume, the tissue slice remains a useful reference for comparison. In the OA map of the GPe trajectory, we observe the cerebral cortex followed by a moderately birefringent WM region, which is then followed by a highly birefringent structure with an azimuthal angle of 0 deg or 180 deg (it is represented as two separate structures due to the representation as *en face* map). The high retardance value of this structure in the GPe trajectory suggests a strong alignment between the plane perpendicular to the probe beam direction and the three-dimensional orientation of the fiber bundles of this structure. Upon exploring the fiber tracts surrounding the basal ganglia nuclei, particularly the GPe, we identified this strongly birefringent and well-organized fiber tract as the IC. The IC is a dense bundle of fibers located between the thalamus and CD medially, and the lentiform nucleus [pallidum and putamen (PUT)] laterally, as described in Ref. 44. The structure observed in the OA map is most likely the fibers from the posterior region of the anterior limb of the IC, where these fibers lie transversely between the CD and PUT.^45^ Referring to the macaque brain atlas used for the experiment planning and target coordinate calculations,^37^ the OA map aligns well with the brain atlas and the information from the literature. The literature states that fiber tracts in the anterior limb of the IC consist of anteroposteriorly directed frontopontine and thalamocortical projections.^46^ This fully aligns with the reconstructed OA orientation, which appears to lie anteroposteriorly in the brain, and, hence, circumferentially to the catheter on the two sides. There is a similar analogy with the tissue photograph, though it captures only a portion of the imaged volume compared with the OA map. These findings are particularly valuable because this fiber tract is not visible in the T1w MRI of the same GPe trajectory, likely due to the MRI’s lower resolution and contrast. This highlights the advantages of PS-OCT in revealing fine structural details that MRI may miss. Figures 4(b) and 4(c) present the tissue photograph along with the OA map and the atlas of the CD and STN imaged trajectories. As expected, the imaged trajectory of the CD reveals cerebral cortex followed by subcortical WM fibers, with no detectable traces of the IC. This observation is consistent with both the tissue photograph and the brain atlas. Notably, near the tail of the CD, distinct fiber bundles are identified in the brain atlas (indicated by the red outline). These bundles are also visible in the OA carpet view, exhibiting slight variations in orientation based on the colormap. The OA carpet view of the STN trajectory contains the same rich information, revealing highly birefringent WM structures surrounding the STN. These fiber tracts might correspond to H fields of Forel, which research suggests could be targeted to treat dystonia and PD.^47^^,^^48^ Such structures could serve as valuable landmarks for neurosurgical guidance with catheter-based PS-OCT.

## 4 Discussion

In this study, we explored the feasibility of using catheter-based PS-OCT as an intraoperative imaging tool for guiding DBS neurosurgery in a nonhuman primate. By comparing PS-OCT imaging with MRI, we assessed the ability of PS-OCT to visualize brain structures critical for stereotactic neurosurgical procedures. Our findings indicate that PS-OCT provides high-resolution anatomical information that is not accessible through conventional MRI, demonstrating its potential as a valuable supplementary tool for neurosurgical navigation. In fact, the WM structures identifiable by PS-OCT often serve as key landmarks for DBS targeting or even serve as direct targets for stimulation.^49^^,^^50^

Nowadays, dMRI and tractography are increasingly recognized as valuable tools for pre-surgical planning.^12^^,^^50^^,^^51^ One clinical application of dMRI is in patients with tremor undergoing VIM thalamic DBS. As the VIM is not directly visible on standard 3T MRI,^52^ its targeting relies on indirect measurements and atlas-based references. Although advanced MRI techniques, such as dMRI for mapping fiber tracts in three dimensions, fast gray matter acquisition T1 inversion recovery for contrast enhancement, and ultrahigh-field 7T MRI for improved resolution, aid in visualizing critical neurosurgical targets,^12^^,^^53^^–^^55^ access to these modalities remains limited to specialized centers, reducing their practicality in more general clinical settings. By contrast, PS-OCT offers fast imaging with micrometer-scale resolution, providing access to tissue structures equivalent to those obtained with dMRI, making it particularly valuable for intraoperative decision-making. Surgeons would be able to receive feedback on the structural details of brain tissues during DBS procedures, facilitating precise targeting and localization of brain structures. The ability to visualize fine tissue structures during neurosurgery could significantly improve surgical accuracy and reduce the risk of errors due to misalignment or incomplete targeting. Therefore, the potential of PS-OCT to provide equivalent information to dMRI, serving as a complementary technique for DBS planning and intraoperative use, is significant. However, one current limitation of PS-OCT is its inability to capture full three-dimensional fiber orientations as the technique primarily measures azimuthal OA orientation within the plane perpendicular to the probe beam direction. In other words, the birefringence observed by the probing beam is influenced by how its propagation direction aligns with the three-dimensional OA of the tissue. Future strategies to retrieve the full 3D orientation with out-of-plane angles^56^ could further enhance the applicability of PS-OCT in neurosurgery. Obtaining three-dimensional OA orientation has been demonstrated in polarized light imaging^26^ and benchtop PS-OCT systems^57^ but not yet in a catheter-based PS-OCT system.

As shown earlier, PS-OCT successfully visualized the IC, a critical WM structure relevant to multiple DBS applications, such as OCD,^4^^,^^6^^,^^7^ which may be treated by stimulating the IC. As previously mentioned, the IC can also serve as a landmark to verify the correct DBS trajectory. In our GPe trajectory, the PS-OCT orientation map revealed highly birefringent fiber tracts most likely corresponding to the posterior region of the anterior limb of the IC. This level of structural details was not discernible in the corresponding MRI scans, highlighting the limitations of MRI contrast when it comes to the level of details needed for precise neurosurgical guidance. Despite these advantages, some discrepancies were observed among MRI, PS-OCT, and post-mortem tissue sections. These differences likely result from a combination of factors, including tissue shrinkage, individual anatomical variations from standard brain atlases, the MRI partial volume effect (where low resolution causes signal averaging by mixing different tissue types within a voxel and reduces contrast between them),^43^ and minor misalignment during MRI-PS-OCT co-registration. In particular, co-registration inaccuracies may stem from experimental factors such as the mechanical properties of the tools used for trajectory marking. Although the vitamin E–filled capillary tubes used to mark the imaging path are relatively rigid, the PS-OCT probe itself, although sufficiently stiff over the distal 20 mm, is somewhat more flexible and susceptible to slight bending during insertion. Although the probe and head were securely fixed in the stereotactic frame and care was taken to avoid deformation, even minor deviations could contribute to co-registration errors and anatomical mismatch. Future improvements could include leaving the catheter sheath in place during MRI marking and filling it with vitamin E or using a more rigid, glass-like sheath to better preserve trajectory geometry. In addition, using subject-specific MRI instead of a population-based template may further enhance anatomical correspondence. Although it is difficult to quantify the misalignment precisely, we expect the discrepancies to be within a sub-millimeter range, likely below the limits that currently impact neurosurgical accuracy. In addition, variations in cortical thickness, as well as the effects of flattening and curvature of surface cortex, may have contributed to the structural differences observed.^58^ Another consideration is the probe design. Our current catheter has a diameter of 2.3 mm, which is larger than standard DBS electrodes that have diameters of ∼1.4  mm. Intravascular OCT routinely uses smaller catheters with an outside diameter of ≤800  μm.^59^^–^^61^ Developing a smaller PS-OCT probe with dimensions comparable to DBS leads would be straightforward and facilitate direct integration into existing surgical workflows.^62^ Although the SMAC-MRI6 template^41^ provided an important reference for co-registration, its lack of raw dMRI data limited direct comparisons between dMRI-based fiber tractography and PS-OCT fiber orientation as the extracted information is fundamentally equivalent. Specifically, fractional anisotropy in dMRI corresponds to the birefringence-derived retardance in PS-OCT, both reflecting tissue anisotropy. In addition, the principal direction of diffusion in dMRI aligns with the OA orientation in PS-OCT. Notably, as discussed previously, dMRI offers a three-dimensional representation, whereas the OA orientation in PS-OCT is inherently confined to a two-dimensional, in-plane measurement. Therefore, the next step of the project could be to establish a direct comparison between catheter-based PS-OCT and dMRI by acquiring corresponding dMRI data or leveraging an appropriate template. This would enable a more comprehensive validation of PS-OCT contrast compared with established diffusion-based tractography methods, further refining its potential for neurosurgical guidance. Finally, integrating PS-OCT could improve surgical precision by providing intraoperative microstructural feedback, enhancing target localization, and reducing dependence on indirect approaches. Although true real-time PS-OCT processing is not yet available, current implementations allow for intraoperative analysis within a few minutes per trajectory, which remains compatible with typical neurosurgical workflows. Future research should prioritize *in vivo* validation, correlation between catheter-based PS-OCT and dMRI, development of real-time processing and visualization algorithms, further probe refinement, and integration into neurosurgical workflows to maximize its clinical utility.

## 5 Conclusion

This study highlights the potential of catheter-based PS-OCT as an effective intraoperative imaging tool for guiding DBS neurosurgery. By providing high-resolution structural and birefringence information, PS-OCT complements MRI, offering additional insights into brain structures critical for precise neurosurgical targeting. The information obtained from PS-OCT aligns well with MRI and could make PS-OCT a valuable supplementary tool to integrate into widely used MRI methods in DBS procedures. PS-OCT’s ability to visualize WM structures, such as the IC fibers’ orientations, may enhance targeting accuracy and provide real-time feedback that MRI cannot achieve. Although PS-OCT offers superior resolution, future advancements could broaden its applicability, such as the ability to capture full three-dimensional fiber orientations. Further comparisons with dMRI and *in vivo* validation will be crucial to refining PS-OCT’s role in neurosurgical guidance. Ultimately, clinical trials and real-time application in surgical settings will be necessary to validate its potential benefits for improving patient outcomes, enhancing precision, reducing reliance on indirect methods, and achieving better results in DBS procedures.

## Data Availability

An open-source MATLAB package for the reconstruction of absolute depth-resolved tissue birefringence is available at https://github.com/CBORT-NCBIB/GuideStarOpticAxisReconstruction. Other data and processing scripts are available upon request.
